# Spliceozymes: Ribozymes that Remove Introns from Pre-mRNAs in Trans

**DOI:** 10.1371/journal.pone.0101932

**Published:** 2014-07-11

**Authors:** Zhaleh N. Amini, Karen E. Olson, Ulrich F. Müller

**Affiliations:** Department of Chemistry and Biochemistry, University of California San Diego, La Jolla, California, United States of America; The Scripps Research Institute, United States of America

## Abstract

Group I introns are pre-mRNA introns that do not require the spliceosome for their removal. Instead, they fold into complex three-dimensional structures and catalyze two transesterification reactions, thereby excising themselves and joining the flanking exons. These catalytic RNAs (ribozymes) have been modified previously to work in trans, whereby the ribozymes can recognize a splice site on a substrate RNA and replace the 5′- or 3′-portion of the substrate. Here we describe a new variant of the group I intron ribozyme from *Tetrahymena* that recognizes two splice sites on a substrate RNA, removes the intron sequences between the splice sites, and joins the flanking exons, analogous to the action of the spliceosome. This ‘group I spliceozyme’ functions in vitro and in vivo, and it is able to mediate a growth phenotype in *E. coli* cells. The intron sequences of the target pre-mRNAs are constrained near the splice sites but can carry a wide range of sequences in their interior. Because the splice site recognition sequences can be adjusted to different splice sites, the spliceozyme may have the potential for wide applications as tool in research and therapy.

## Introduction

Group I introns are intervening sequences in pre-mRNAs that do not require the spliceosome for their removal [1]. Instead, they fold into three-dimensional structures and catalyze two transphosphorylation reactions resulting in their excision from the primary transcript and the joining of their flanking exons [1], [2], [3]. The biochemistry of group I intron ribozymes has been studied extensively (for review, see [4], [5]) and crystal structures have been reported for group I introns from three different species [6], [7], [8].

Group I intron ribozymes have been engineered to catalyze several non-native reactions. For example, they can be converted from the natural cis-splicing format into a trans-splicing format, by removing the ribozyme 5′-exon and replacing it with a short substrate recognition sequence [9], [10]. This substrate recognition sequence base pairs to a complementary target site on the substrate RNA, thereby specifying the splice site [11], [12]. In the subsequent reaction, the ribozyme replaces the 3′-portion of the substrate RNA with its own 3′-exon; this has been demonstrated in vitro and in bacterial and mammalian cells [10], [13], [14]. In addition to replacing the substrate 3′-portion by the ribozyme 3′-exon, group I intron ribozymes are also able to replace the substrate 5′-portion by the ribozyme 5′-exon. In this case, the recognition of the splice site is more complicated, using the concerted action of the ribozyme 5′-terminus and 3′-terminus [15], [16].

When the two types of reactions - replacement of the substrate 3′- and 5′-portion - are combined, group I intron ribozymes can facilitate trans-excision reactions - the removal of an internal RNA segment and the joining of the flanking exons [17]. This trans-excision reaction relies upon three recognition sequences, consisting of the formation of the P1, P9.0, and P10 helices between substrate and ribozyme [16], [18]. These trans-excision ribozymes were shown to excise fragments of up to 28 nucleotides from short substrates in vitro [17], and remove single nucleotides from full-length mRNA in *E. coli* cells [19]. No evidence has indicated that longer fragments can be excised in cells. To obtain ribozymes that can efficiently excise long internal substrate fragments from full length mRNAs under physiological conditions, a different splice site recognition principle was used here to recognize the 5′- splice site and the 3′- splice site with two separate structural elements, the P1 and P9.2 helix, respectively.

**Figure 1:**
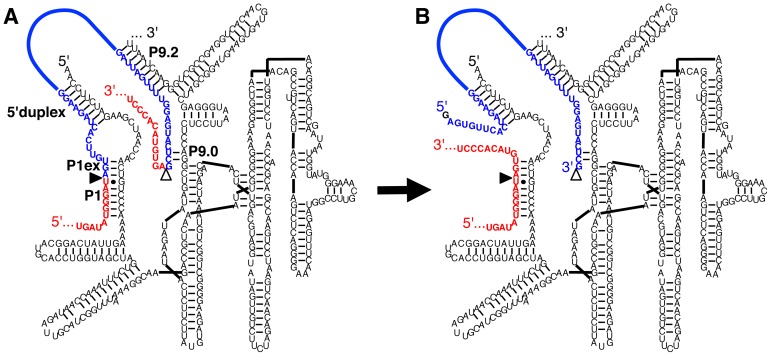
Secondary structure of the spliceozyme, based on the group I intron ribozyme from *Tetrahymena*. (**A**) Before the reaction, the substrate consists of two exons (red) and an intervening intron (blue). The spliceozyme (black) uses its 5′-terminus to form the P1 duplex, the P1 extension (P1ex), and the 5′-duplex to position the substrate 5′-splice site (filled triangle). The ribozyme 3′-terminus forms the P9.2 duplex and the P9.0 duplex to position the substrate 3′-splice site (empty triangle). (**B**) After two transphosphorylation reactions the 5′-exon and the 3′-exon of the substrate are joined. The 5′-terminus of the intron is capped by an exogenous G (black) and the 3′-terminus of the intron is liberated. The sequences correspond to the removal of an intron from splice site 258 of the *CAT* pre-mRNA used in this study. The internal sequence of the intron is drawn as a bold blue line. Note that a hairpin terminator is added to the 3′-terminus for all reactions in cells. The secondary structure of the ribozyme is based on [58] with the alteration that the P4-P6 domain was positioned on the right side for clarity.

Here we demonstrate that a new variant of the group I intron from *Tetrahymena* can remove introns from a full-length mRNA in trans. The ribozyme differs from the described trans-excision ribozymes by using its 3′-terminal P9.2 helix for recognition of the 3′-splice site (Figure 1), which leads to the efficient excision of introns with a length of 100 nucleotides. The excision is accurate in vitro and in *E. coli* cells, and tolerates many sequences within the intron. We termed this RNA ‘spliceozyme’ because it is a ribozyme that functions like a spliceosome. After further developments, the spliceozyme may serve as a versatile tool to remove internal RNA sequences for applications in research and in therapy.

## Results

### Design of the Spliceozyme

The spliceozyme described here is based on the group I intron ribozyme from *Tetrahymena* (Figure 1). For recognition of the 5′-splice site, the substrate sequence immediately adjacent to the splice site was recognized by forming the P1 helix and the P1 extension helix, followed by an internal loop and a duplex at the ribozyme 5′-terminus [12], [13], [20], [21]. The ribozyme design with only a P1 duplex was labeled P1; the design with a P1 duplex and a P1 extension was labeled P1ex, and the design that included all elements of the extended guide sequence (P1 helix, P1 extension, internal loop, and 5′-duplex) was labeled EGS. For recognition of the substrate 3′-splice site, the P9.2 helix was designed to form in trans, and was truncated to 9 base pairs. The substrate for the trans-splicing reactions described here was the 678-nucleotide long mRNA encoding the enzyme chloramphenicol acetyl transferase (CAT), with an intron of 64 or 100 nucleotides in length inserted at position 258.

### Spliceozyme activity in vitro

**Figure 2:**
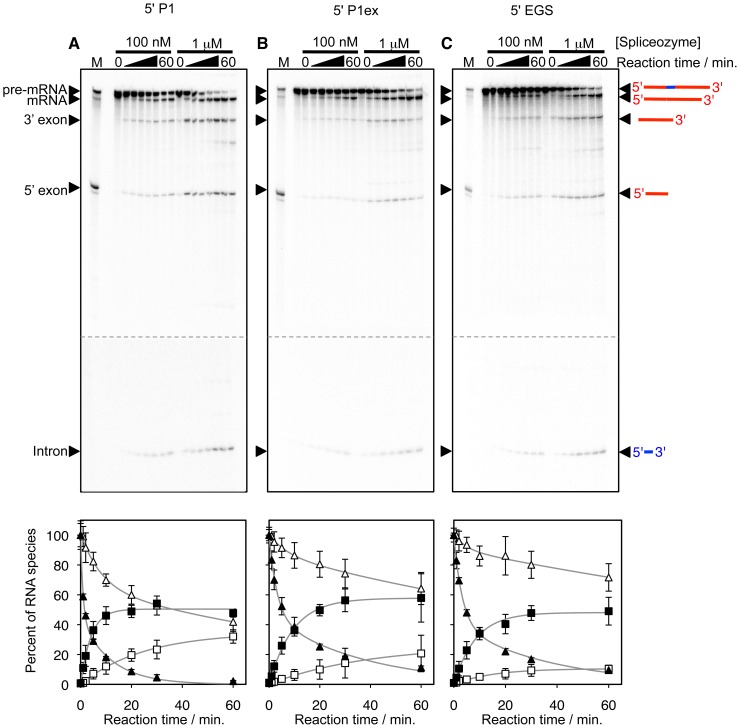
Influence of the spliceozyme 5′-terminus design on the reaction in vitro. The ribozyme 5′-terminus ends in (**A**) a P1 duplex, (**B**) a P1 extension, or (**C**) a 5′-duplex (see Figure 1). The corresponding constructs are labeled as 5′ P1, 5′ P1ex, and 5′ EGS, respectively. The top panels show autoradiograms of internally radiolabeled splicing products after separation by denaturing polyacrylamide gel electrophoresis. The marker (M) shows the position of pre-mRNA (778 nt), mRNA (678 nt), and 5′-exon (278 nt). The 3′-exon and the intron had a length of 400 nt and 100 nt, respectively. For each spliceozyme construct, two splicing reactions are analyzed with spliceozyme concentrations of 100 nM and 1 µM. Samples were taken at reaction times between 0 and 60 minutes. A schematic of the substrates and reaction products is shown to the right of the image, with exons in red and the intron in blue. Bottom panels show the quantitation of the disappearance of the *CAT* pre-mRNA (triangles) and the appearance of the *CAT* mRNA (squares). Empty symbols correspond to 100 nM spliceozyme, while filled symbols correspond to 1 µM spliceozyme concentration. Grey lines show single-exponential curve fits to the products and double-exponential curve fits to the reaction products. Error bars are standard deviations from three experiments.

To test whether the spliceozyme could function in vitro, the *CAT* pre-mRNA was radiolabeled internally and incubated with spliceozymes targeting the two exon/intron junctions of a 100-nucleotide long intron. Reaction conditions used near-physiological conditions, including 5 mM MgCl_2_ at 37°C, 100 nM *CAT* pre-mRNA, and spliceozyme concentrations of 100 nM and 1 µM. Splicing products of the correct size were detectable after only a few minutes of reaction time (Figure 2, upper panels). Quantitation of the signals showed that within five minutes, 3–7% and 23–36% of the pre-mRNA were converted to a splicing product of the correct size by 100 nM and 1 µM spliceozyme, respectively (Figure 2, lower panels). The ribozyme with a P1 5′-terminus reacted approximately 2.5-fold faster than the other ribozymes (0.25 min^−1^ versus 0.09–0.12 min^−1^, respectively), each with a P9.2 duplex length of 9 base pairs (compare Figures 2A, B, and C). This suggests a possible inhibitory role of extensions at the 5′-terminus in the P1ex and EGS constructs, and is consistent a previous suggestion that the ribozyme 5′-structure could indeed be partially inhibitory for trans-splicing [22].

To determine if the products contained the correct splicing junction, the reaction products were subjected to RT-PCR, specifically amplifying sequences that contained both the 5′-exon and the 3′-exon of the *CAT* mRNA. Cloning and sequencing showed the correct exon junction sequence in ten out of ten trans-splicing products (data not shown). These results demonstrated that a spliceozyme using its 5′-terminus for recognition of the 5′-splice site and its 3′-terminus for recognition of the 3′-splice site via formation of the P9.2 helix was able to efficiently and accurately remove the intron from a pre-mRNA in vitro.

Between 8% and 46% of the substrate molecules lost their 5′-exon and were thereby converted to side products within one hour (Figure S1A). The differences were correlated with two factors: spliceozyme concentration and design of the spliceozyme 5′-terminus. First, a spliceozyme concentration of 1 µM led to approximately 4-fold greater release of the 5′-exon compared to the same constructs at a ribozyme concentration of 100 nM, likely a result of more reaction events generating more side reaction events. Second, in constructs differing in the ribozyme 5′-terminus (all with a P9.2 duplex length of 9 base pairs) approximately two-fold higher 5′-exon release was observed in the absence of a P1 extension duplex (construct P1) compared to the other two constructs that contained a P1 extension duplex (P1ex, EGS). One possible explanation for this behavior is that the ribozyme 5′-terminus forming the P1 extension helps position the 3′-splice site at the catalytic site by forming the P10 helix [23]; this helix formation may therefore shorten the time between the first and the second catalytic step of splicing, during which the 5′-exon can escape from the spliceozyme. At 1 µM spliceozyme concentration additional side products are detectable in Figure 2; the identity of the corresponding side products is currently unclear.

### Influence of the P9.2 duplex length on spliceozyme activity in vitro

**Figure 3:**
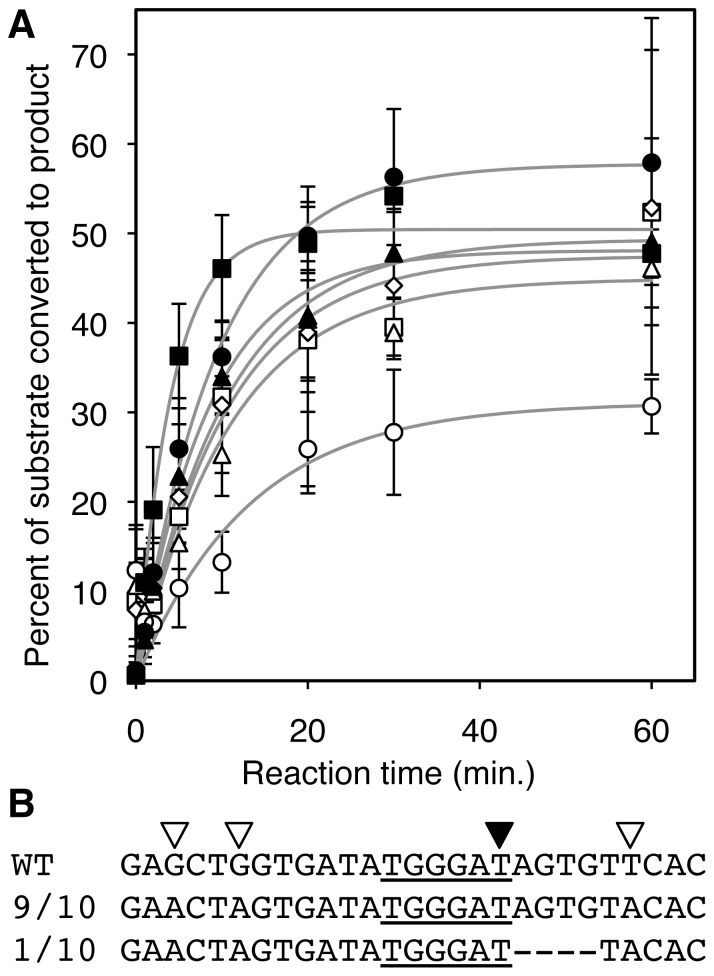
Effect of substrate recognition sequences on product formation. (**A**) Kinetics of spliceozyme splicing in vitro, measured as the molar percentage of the initital substrate concentration (*CAT* pre-mRNA) converted to product (*CAT* mRNA). The reactions were analyzed as in Figure 2. Data from spliceozymes with a 3′-terminal P9.2 helix of 9 base pairs are shown with filled symbols, including spliceozyme 5′-termini of a 5′-duplex (triangles), P1 extension helix (circles), and P1 helix (squares). Empty symbols denote results from spliceozymes terminating in a P1 helix at the 5′-terminus, and the 3′-terminal P9.2 helix truncated to 8 base pairs (diamonds), 7 base pairs (squares), 6 base pairs (triangles), and 5 base pairs (circles). Error bars correspond to standard deviations from three experiments. (**B**) Sequences of splicing junctions resulting from RT-PCR, cloning and sequencing of trans-splicing products from the reaction with a P9.2 helix of 5 base pairs and a P1 helix at the 5′-terminus. Note that the wild type sequence (WT) differs from the splicing products in three silent mutations (empty triangles), which confirmed that the sequences were splicing products and not a contamination by the wild type gene. Nine out of ten sequences showed the sequence expected from correct splicing at the 3′-terminal G, whereas one sequence indicated that the guanosine four nucleotides downstream of the intended 3′-splice site was used instead. The sequence participating in the P1 helix is underlined, containing the 5′-splice site (filled triangle).

Truncation of the P9.2 helix from 9 base pairs to 8, 7, or 6 base pairs did not significantly affect spliceozyme efficiency (Figure 3A). Only when the length of the P9.2 helix was reduced to 5 base pairs was a lower fraction of substrate converted to product than ribozymes with a P9.2 helix of six to nine base pairs (31% versus 45%–58%, respectively). This is consistent with the finding that duplexes shorter than 7 base pairs are below the optimal length for the specific recognition of target sites [24]. The length of the P9.2 duplex did not strongly influence the amount of 5′-exon release (Figure S1B).

To estimate the effect of truncating the P9.2 helix on splicing accuracy, ten splice site junctions were determined by reverse transcription, cloning and sequencing of the splicing products resulting from a P1 helix of 6 base pairs and a P9.2 helix with 5 base pairs (Figure 3B). Nine out of ten splice site junctions had the correct sequence. One sequence gave evidence for an event of aberrant splicing, in which the guanosine four nucleotides downstream of the correct guanosine was chosen as 3′-splice site. In contrast, ten out of ten sequences showed the correct product when the 3′-splice site was recognized by a 9 base pair helix (data not shown). This suggested that a P9.2 duplex length of 5 base pairs may be too short for reliable recognition of the correct 3′-splice site, consistent with earlier considerations on substrate recognition helices [24]. Together, the truncation experiments at the ribozyme 5′- and 3′-terminus suggested that a P1 extension at the 5′-splice site reduced the loss of 5′-exons, and that the 3′-splice site required a P9.2 duplex with at least 6 base pairs.

### Spliceozyme activity in cells

**Figure 4:**
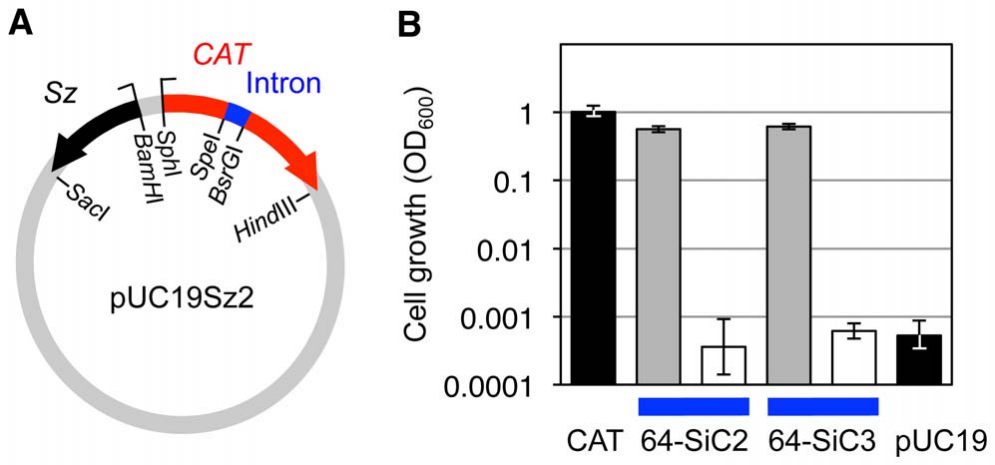
Spliceozyme activity in *E. coli* cells. (**A**) Schematic of the plasmid used for the expression of spliceozyme (Sz; black) and *CAT* pre-mRNA (*CAT;* red) containing an intron (blue) in *E. coli* cells. Restriction sites used in cloning of the plasmid are indicated. (**B**) Quantitation of *E. coli* cell growth on LB-agar plates containing 8 µg/mL chloramphenicol, by measuring the A_600_ of cell suspensions from washing the plates after incubation. The black column on the left shows the A_600_ from cells that express the wild type *CAT* gene without intron. Grey columns denote the A_600_ resulting from plasmids containing a *CAT* gene with an intron and wild-type spliceozymes. White columns denote the A_600_ from the same constructs as in the grey columns but with six mutations in the catalytic core of the spliceozymes. The black column on the right denotes the A_600_ resulting from cells containing the pUC19 plasmid without spliceozyme or *CAT* gene. Two different intron sequences with a length of 64 nucleotides were inserted into plasmid pUCSz2 for this assay (64-CiC2 and 64-SiC3; note that both of these introns were selected for their efficient removal by the spliceozyme; see materials and methods). Note the logarithmic scale for the A_600_. Error bars are standard deviations from three biological samples.

To assess the activity of the spliceozyme in cells, a plasmid encoding the spliceozyme and a *CAT* pre-mRNA with a 64-nucleotide long intron was generated, and transformed into *E.coli* cells (Figure 4A). In order for bacterial resistance to chloramphenicol to be conferred, it is necessary for the spliceozyme in the cells to process *CAT* pre-mRNA to *CAT* mRNA, which is then translated to the CAT enzyme. The CAT enzyme acetylates chloramphenicol with acetyl-coA as acetyl donor [25], rendering chloramphenicol unable to inhibit the ribosome [26], thereby allowing *E. coli* cells to grow in the presence of chloramphenicol. Indeed, *E.coli* cells transformed with the spliceozyme construct were able to grow on medium containing 8 µg/mL chloramphenicol (Figure 4B; grey columns), at levels similar to the growth mediated by *CAT* mRNA without an intron (Figure 4B, black column on the left). To test whether this growth was a result of spliceozyme-mediated catalysis, the catalytic center of the spliceozyme was inactivated by mutations. A mutation at the guanosine binding site (G264A) was found previously to strongly reduce ribozyme activity while maintaining the overall ribozyme fold [27], [28], [29]. Because the remaining weak activity appeared to generate some bacterial growth (data not shown) the ribozyme was further inactivated with a total of six mutations (A261U, C262A, A263G, G264A, A265U, and C266A). The modified construct did not mediate detectable bacterial growth (Figure 4B, white columns) above the background level of the assay (Figure 4B, black column on the right), approximately 1/1,000 of the signal from the functional spliceozyme. Two different introns in the pre-mRNA resulted in similar levels of growth with active spliceozyme and no growth with inactive spliceozyme (constructs 64-SiC2 and 64-SiC3; Figure 4B). Note that these two intron sequences were not arbitrarily chosen but were the result of a selection (see below). To estimate the splicing accuracy of the spliceozyme in cells total RNA was isolated and used to determine the sequence of splice junctions in the *CAT* mRNA as described for the in vitro reaction. Ten out of ten trans-splicing products had the correct sequence (data not shown). These results demonstrated that catalysis by the spliceozyme removed the intron from a pre-mRNA in *E. coli* cells, and did so accurately and efficiently enough to generate an antibiotic resistance phenotype.

### Sequence requirements of the intron

**Figure 5:**
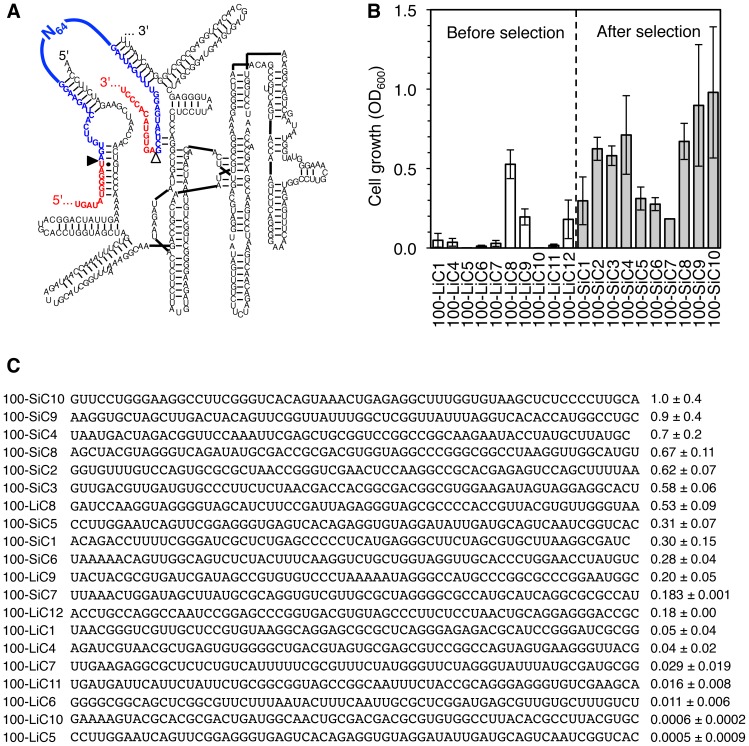
Effect of the internal intron sequence on spliceozyme activity in *E. coli* cells. (**A**) Secondary structure of the spliceozyme (black), with the substrate exons in red and the position of the randomized N_64_ sequence indicated in the internal region of the intron (blue). (**B**) Quantitation of *E. coli* cell growth on LB-agar plates containing 10 µg/mL chloramphenicol. The resulting A_600_ is given for 20 clones, ten of which were chosen before selection on LB-chloramphenicol plates (white columns), and ten of which were chosen after this selection step (grey columns). The name of each clone is given below, with ‘L’ indicating clones from the unselected library and ‘S’ indicating selected clones, 100 denoting the length of the intron, the letter i indicating that the internal region of the intron was randomized, and the number after ‘C’ denoting the clone number. Error bars are standard deviations from three experiments. (**C**) Sequences of 20 cloned intron sequences, sorted according to their activity in cells. The left column lists the clone names, using the same nomenclature as in (B). The middle column shows the internal sequence of that clone that resulted from the N_64_ library, which was inserted between the constant regions of the intron (see (A)). The right column shows the growth activity, measured as A_600_ of cell suspensions that resulted from washing LB-growth plates containing chloramphenicol, relative to growth on medium without chloramphenicol.

To determine whether the spliceozyme required specific sequences within the intron of the *CAT* pre-mRNA, two selection experiments were performed with a 100-nucleotide long intron. In the first selection experiment, the internal region of the intron between the substrate recognition sequences was randomized (N_64_; Figure 5A; clone names contain an i for ‘internal’). A library of plasmid constructs was generated that was identical to the construct described above but differed in the length of the intron (100 nucleotides) and the randomization of the 64 internal nucleotides of the intron sequences. *E. coli* cells were then transformed with this plasmid library. Ten randomly chosen clones from this *E. coli* library were tested for growth on LB plates with chloramphenicol (10 µg/mL). Eight out of ten clones mediated detectable growth, and three of them mediated moderate growth (Figure 5B, white columns; clone names contain an L for ‘library’) with efficiencies similar to an additional ten clones that were selected from the library for efficient growth on LB-chloramphenicol medium (Figure 5B, grey columns; clone names contain an S for ‘selected’). When the sequences of all 20 introns were correlated with the mediated chloramphenicol resistance, no sequence pattern could be identified that was associated with increased activity, based on a comparison of their sequences, predicted secondary structures, and predicted self-folding energies [30] (Figure 5C). A similar selection was performed with a 64-nucleotide long intron containing a randomized sequence of 28 nucleotides. This experiment also gave rise to highly efficient introns, and similarly did not reveal favored patterns for the intron sequences (data not shown). Although neither of these experiments covered the complete sequence space of the randomized N_64_ and N_28_ sequences, this was not necessary to estimate the frequency of beneficial intron sequences within random libraries. The results revealed that the spliceozyme efficiency varied significantly with the internal sequence of the pre-mRNA intron, and that approximately 30% of sequences from a random library constituted a favorable internal intron sequence for processing by the spliceozyme.

**Figure 6:**
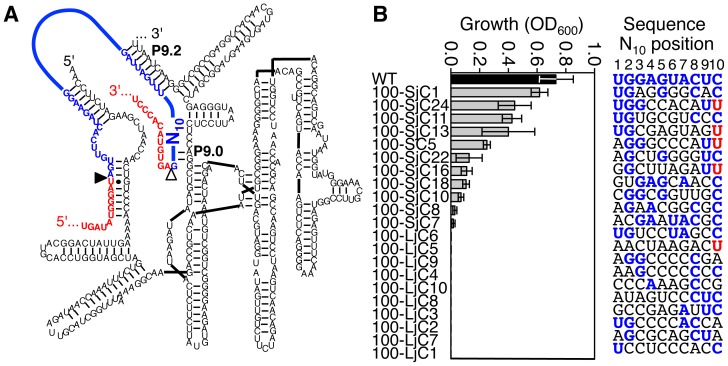
Effect of the 3′-terminal intron sequence on spliceozyme activity in *E. coli* cells. (**A**) Secondary structure of the spliceozyme (black), with the substrate exons in red, and the position of the randomized N_10_ sequence in the 3′-terminal region of the intron (blue). The helices P9.0 and P9.2 are labeled. (**B**) List of 20 analyzed clones, sorted according to the resulting activity in cells. The clone name is given on the left, the growth on LB-agar plates containing chloramphenicol is shown as horizontal columns, and the N_10_ sequence is given on the right. Clone names containing ‘L’ indicate the unselected library, while ‘S’ indicates a selection step with chloramphenicol. The letter ‘j’ denotes that the randomized region is located near the 3′-terminus of the *CAT* mRNA intron. The growth was measured on plates containing 10 µg/mL chloramphenicol and normalized to growth on medium without chloramphenicol. The sequence at the top (WT) corresponds to the wild type sequence of the *Tetrahymena* ribozyme. Nucleotides that are identical with the wild type sequence are colored in blue. The position 10 tolerates a U, which is consistent with its base-pairing role, and is colored in red. Errors are standard deviations from three experiments.

The second selection experiment with an intron of 100 nucleotides tested the sequence requirement for the ten nucleotides between the 3′-splice site and the P9.2 helix (N_10_; Figure 6A). These ten nucleotides were randomized similar to the previous selection, and the corresponding plasmid library was transformed into *E.coli* cells. When ten randomly chosen clones from this *E.coli* library were tested for growth on medium containing 10 µg/mL chloramphenicol, none showed detectable growth (Figure 6B, clone names contain an L for ‘library’). Growth was also measured for ten clones selected from the small fraction of the library that mediated growth on LB-chloramphenicol medium (Figure 6B, clone names contain an S for ‘selected’). When the sequences of these clones were sorted according to the level of mediated growth a correlation between splicing activity and the identity of nucleotides 1, 2, and 10 within this region became apparent (Figure 6B, right). The five most efficient sequences (Figure 6B, column graph) were used to characterize the sequence requirement in this region. At position 1, four of these five sequences carried a U, consistent with the wild type ribozyme sequence (compared to 3/10 sequences from the selected library). At position 2, all five sequences carried a G, consistent with the wild type, and in contrast to the pre-selected pool (4/10 sequences). This is consistent with the role of nucleotide 2 in base pairing to a C adjacent to the P9.0 helix [31] in the wild type ribozyme. At position 10, all active sequences carried a C or a U, consistent with the wild type C, and different from the pre-selected pool (6/10 sequences). This is consistent with the role of nucleotides 9 and 10 in forming the P9.0 helix [32], [33], [34]. At position 9, the presence of four U's in the 7 most active clones would be consistent with a weak selection at this position. No significant enrichment was observed at other positions. Together, these results suggested that the 10-nucleotide sequence between the P9.2 helix and the 3′-splice site was constrained to specific nucleotides in at least 3 positions.

### Efficiency of the spliceozyme in cells

**Figure 7:**
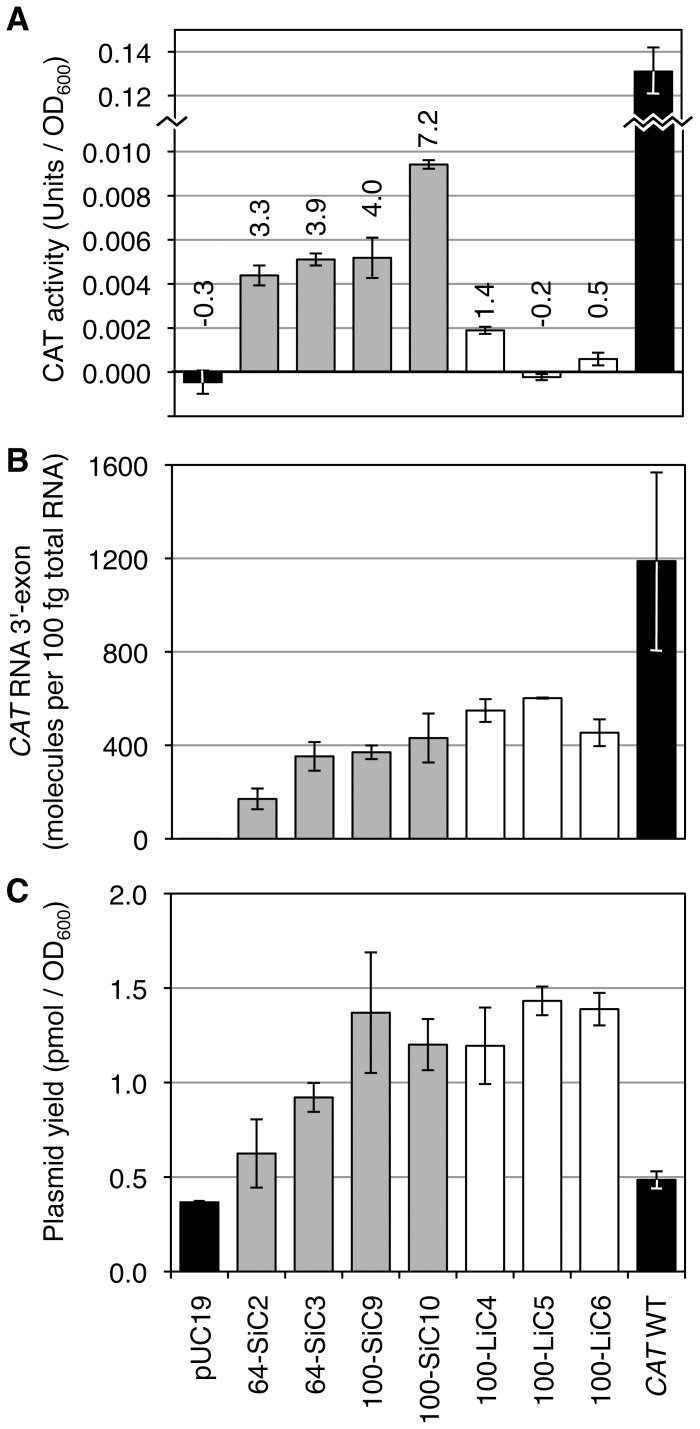
Quantitation of CAT enzyme activity, *CAT* RNA level, and plasmid level in *E. coli* cells. (**A**) Units of CAT activity detected in *E. coli* cell extract, containing different plasmid constructs, as indicated below the column graphs. No detectable CAT activity was found when *E. coli* cells contained the plasmid pUC19 (left black column; -0.00045±0.00053 units per OD_600_). Grey columns show the CAT activity of constructs expressing a spliceozyme and a *CAT* pre-mRNA containing an intron that mediated efficient bacterial resistance to chloramphenicol. White columns show the CAT activity mediated by introns that led to poor or no bacterial resistance to chloramphenicol. Construct names starting with 64 or 100 label the length of the intron in the *CAT* pre-mRNA gene, ‘S’ denotes that the clone was selected for activity on medium with chloramphenicol, ‘L’ denotes that the clone was chosen from an un-selected library, ‘I’ denotes that the library for this selection was randomized in the internal region of the intron, and the number after C is the clone number. *CAT* WT denotes a construct without spliceozyme, and in which the CAT gene did not contain an intron, resulting in 0.13±0.01 units of CAT activity per OD_600_. The percent of CAT activity relative to the *CAT* WT construct is noted above each column. (**B**) Level of *CAT* RNAs estimated by RT-qPCR of the *CAT* RNA 3′-exon. This assay measures the sum of *CAT* pre-mRNA and *CAT* mRNA. (**C**) Molar amount of plasmid isolated from logarithmically growing *E. coli* cells. Note that the unit OD_600_ is used as an absolute value, corresponding to the cells in a volume of 1 mL cell suspension with OD_600_ = 1. For all graphs shown in Figure 7, error bars denote standard deviations from three biological replicates.

To obtain a measure for the efficiency of the spliceozyme in *E. coli* cells the amount of active CAT enzyme in *E. coli* cell extract, the formation of which requires the correct processing of *CAT* pre-mRNA, was quantified (Figure 7A). CAT activity was measured by an assay utilizing Acetyl-CoA and DTNB (5,5-Dithio-bis(2-nitrobenzoic acid)) [35]. Four constructs expressing spliceozyme and *CAT* pre-mRNA with introns that had previously mediated efficient growth on chloramphenicol containing medium resulted in CAT levels between 0.004 and 0.010 units of CAT per OD_600_ of cells (grey columns). This corresponded to 3.3% and 7.2% of the activity generated by a construct expressing *CAT* mRNA without an intron, in the absence of spliceozyme expression (0.13 units of CAT per OD_600_ of cells; black column on the right). In contrast, three constructs expressing spliceozyme and *CAT* pre-mRNA introns that mediated only poor growth or no growth showed significantly lower CAT activity in the bacterial extract (white columns). This confirmed that spliceozyme-facilitated CAT activity mediated the bacterial chloramphenicol resistance observed in the previous experiments.

To test whether the variation in CAT activity between different intron sequences was mediated by changes in the *CAT* pre-mRNA/mRNA expression level, total RNA was isolated from each of the bacterial constructs, and *CAT* pre-mRNA/mRNA 3′-exons were quantified by RT-qPCR (Figure 7B). Resulting 3′-exon levels indicated that RNA expression levels were not higher for efficient introns (grey columns) than for inefficient introns (white columns), thereby excluding the possibility that the CAT activity differences were caused by differences in the *CAT* RNA expression levels. Similarly, plasmid expression levels (Figure 7C) of efficient introns (grey columns) were not higher than those of inefficient introns (white columns), excluding the possibility that these introns acted by affecting the plasmid copy numbers. It is interesting to note that the lowest plasmid copy number was observed with pUC19 (no expression of pre-mRNA or spliceozyme), with a slightly higher expression for the construct that expressed the *CAT* mRNA, and even higher copy numbers for plasmids that expressed both *CAT* pre-mRNA and spliceozyme. This finding, however, does not affect the conclusion regarding modulation of plasmid copy numbers or RNA expression levels the *CAT* pre-mRNA intron sequences.

Together, these results confirm that catalytic activity of the spliceozymes mediated bacterial resistance to chloramphenicol by processing *CAT* pre-mRNA to *CAT* mRNA, thereby facilitating the translation of active CAT enzyme in bacterial cells. Future improvements may result in spliceozymes that process a larger portion of substrate RNAs, and with less intron sequence dependence, for possible uses as tools in research and therapy.

## Discussion

In this study we demonstrated that variants of the *Tetrahymena* group I intron ribozyme can remove introns in trans from pre-mRNAs by using their 5′-terminus and 3′-terminal P9.2 helix for recognition of 5′- and 3′-splice sites, respectively. These spliceozymes can accurately remove 100-nucleotide long introns from a pre-mRNA, in vitro and in *E. coli* cells, thereby facilitating the expression of functional protein and mediating an antibiotic resistance phenotype. The spliceozymes require specific recognition sequences near the 5′- and 3′-splice sites but tolerate many sequences in the internal region of the intron.

Two short substrate recognition elements in the spliceozyme of five to nine base pairs were sufficient to specify the location of pre-mRNA splicing in *E. coli* cells despite the fact that in principle, any uridylate residue among the many RNAs in *E.coli* cells that is flanked by the complementary sequences could serve as substrate [11], [12]. This observation, however, is consistent with the finding that only a small fraction of all possible splice sites can be used efficiently by trans-splicing ribozymes. This fraction is in the range of ∼3% for the *CAT* mRNA, as estimated by the computation of the total free energy of substrate binding, a good predictor of splice site efficiency [22]. The fraction of efficient splice sites in a cellular environment may be even lower for highly structured and chemically modified RNAs such as the ribosome and tRNAs, which constitute the bulk of cellular RNAs. The precise frequency of off-target effects with the spliceozymes is currently unclear.

Quantitation of CAT activity in *E. coli* cell extract suggested that approximately 5% of the *CAT* pre-mRNA were converted to functional *CAT* mRNA by the spliceozyme, when compared to *CAT* mRNA lacking an intron (Figure 7A). This efficiency is similar to the estimated 3-4% of *CAT* mRNA repaired in a similar bacterial system by a trans-splicing group I intron using only a single splice site [21], [36]. This suggests that the spliceozymes described in this study were similarly efficient as the previously used trans-splicing ribozymes.

Spliceozymes could potentially be employed for several applications in research and therapy. Research applications could include the use of spliceozymes to create and study different splicing isoforms and phenomena such as alternative splicing, exon skipping, or recursive splicing [37], [38], [39], [40]. Spliceozymes could also be used to model specific biochemical steps in the evolution of the spliceosome, which likely originated from a common ancestor with group II intron ribozymes [41], [42], [43], [44], [45], [46]. It is currently unclear how the spliceosome recruited proteins during its evolution, fragmented into multiple RNAs, and developed multiple turnover reactions. The current spliceozyme could therefore serve as a model system to study analogous steps in the evolution from a ribozyme to a dynamic RNP.

Spliceozymes may also have applications in therapy, for example in the correction of aberrant splicing. At least 10% but perhaps 60% of all disease-causing genetic mutations result in aberrant splicing [47], [48], [49]. It may be possible to use spliceozymes for the treatment of these genetic disorders by processing incompletely or incorrectly spliced pre-mRNAs to functional mRNAs. The procedure for this approach would be similar to that envisioned for trans-splicing ribozymes for the replacement of RNA 3′-portions in human cells [13], [50], [51], [52], [53], [54], [55]. For this application to be feasible it would first be necessary to improve the delivery (or in situ expression) of spliceozymes, their efficiency in cells, and minimize potential off-target splicing effects [10], .

## Materials and Methods

### Construction of spliceozyme/*CAT* pre-mRNA expression plasmids

The plasmid expressing the spliceozyme and the *CAT* pre-mRNA (Figure 3A) was based on the previously described pUC19-based expression system [21], which includes a multiple cloning site with SacI, BamHI, SphI and HindIII. The ribozyme expression cassette was inserted between restriction sites SacI and BamHI, and the *CAT* expression cassette between SphI and HindIII, in three steps. First, the *CAT* gene was PCR amplified from the plasmid pLysS (Novagen) as described [21], and prepared for the insertion of an intron at position 258 by introducing three restriction sites through PCR mutagenesis. Specifically, the 3′-PCR primer for amplifying the 5′-exon of the *CAT* gene introduced the silent mutations G243A and G246A to generate a SpeI restriction site, and added a 3′-terminal XmaI site near position 258. The 5′-PCR primer for amplifying the 3′-exon introduced the silent mutation T264A to generate the BsrGI site, and added a 5′-terminal XmaI site near position 258. The PCR products of the two exons were digested with SphI and XmaI (5′-exon) and HindIII and XmaI (3′-exon), then both fragments were ligated into the SphI/HindIII cassette of pUC19b [21] to create plasmid pUCSz1. The sequence with the silent mutations is visible in the sequence of the correctly spliced products (Figure 6B).

Second, the spliceozyme cassette, including the *trc*2 promoter [57] and a hairpin terminator, was generated in successive PCR reactions analogous to another trans-splicing ribozyme [21]. By successively modifying the 5′-terminus of the *Tetrahymena* group I intron gene with 5′-PCR primers, the ribozyme 5′-recognition sequence was adjusted to the new splice site 258 (see Figure 1A), and the *trc*2 promoter and a BamHI restriction site were added. By successively modifying the 3′-terminus by 3′-PCR primers, the 3′-end of the spliceozyme was truncated (see Figure 1A), a hairpin terminator (5′-GCATAACCCCTTGGGGCCTCTAAACGGGTCTTGAGGGGTTTTTTG-3′; stem underlined) was added, and a SacI restriction site was added. The PCR products were then cloned into the BamHI - SacI sites of pUCSz1 to generate pUCSz2.

Third, the intron sequences were inserted into pUCSz2 using the SpeI and BsrGI restriction sites introduced into the *CAT* sequence. Oligonucleotides 5′-gaactagtgatatgggat AGTGTTCACTAGAAGG - N_28_ - GATTAGTTTTGGAGTACTCG agtgtacacc-3′ and 5′-gaactagtgatatgggat AGTGTTCACTAGAAGG - N_64_ - GATTAGTTTTGGAGTACTCG agtgtacacc-3′ were converted to double strands by PCR, digested with SpeI and BsrGI, and inserted into the corresponding sites of pUCSz2 (exon sequences are in lower case, intron sequences in upper case, restriction sites are underlined). This created introns with a total length of 64 and 100 nucleotides, and randomized regions of 28 and 64 nucleotides, respectively. These two plasmid libraries were used in selections for efficient internal intron sequences.

For the selection of intron sequences between the P9.2 helix and the 3′-terminal guanosine (G414), plasmid libraries with ten randomized nucleotides were generated by PCR amplification with a primer that carried the randomized sequence. As PCR template, a clone with an intron length of 100 nucleotides (100-SiC10, Figure 5C) was used. The 3′-PCR primer (5′-ccttgtacactc - N_10_ - AAACTAATCTGCAAGGGGAG-3′) randomized the region of interest, and added the BsrGI site (underlined). All other steps were analogous to the selection of internal intron sequences.

Mutagenic inactivation of the spliceozyme at positions 261–266 was completed by site-directed mutagenesis, using two oligonucleotides that covered 19 bases on each flank of the mutated sequence. The resulting mutations were A261U, C262A, A263G, G264A, A265U, and C266A. The procedure used PrimeSTAR GXL DNA Polymerase (Invitrogen) and the QuickChange™ site-directed mutagenesis protocol developed by Stratagene (La Jolla, CA).

### Selection of efficient intron sequences

Plasmid libraries containing partially randomized introns were transformed into electrocompetent *E. coli* DH5α cells, and the cells were plated onto LB-agar containing 100 µg/mL ampicillin. After incubation at 37°C for 16 hours, the plates were washed with LB medium to generate the bacterial libraries, which could be frozen as glycerol stocks. Bacterial libraries were diluted to an A_600_ of 0.015 with liquid LB medium, and induced with 1 mM IPTG by shaking for 1 hour at 37°C. For the selective step, 100 µL of this culture were plated onto LB-agar plates containing chloramphenicol of the noted concentration, and allowed to grow for 16 hours at 37°C. Individual colonies were selected and analyzed for the sequences and efficiencies of individual introns.

### Spliceozymes and pre-mRNAs for in vitro reactions

Spliceozymes and the *CAT* pre-mRNA substrate were generated by run-off in vitro transcription from PCR products with T7 RNA polymerase, essentially as described [22]. The template for PCR of the spliceozyme, and of *CAT* pre-mRNA, was the plasmid clone pUCSz2_100-SiC10. The PCR of the spliceozyme used 5′-primers that contained the T7 RNA polymerase promoter and truncated 5′-recognition sequences, and 3′-primers that truncated the spliceozyme 3′-termini, as shown in the figures. For PCR amplification of the template for T7 transcription of *CAT* pre-mRNA, the 5′ primer GCGTAATACGACTCACTATAGCAGGAGCTAAGGAAGCTAAAATG and the 3′-primer CGCCCCGCCCTGCCACTCATC were used (the T7 promoter is underlined). Transcriptions were performed at 37°C for 3 hours in 40 mM Tris/HCl pH 7.9, 26 mM MgCl_2_, 5 mM DTT, 0.01% Triton X-100, 2.5 mM spermidine and 2 mM of each NTP. The transcription of *CAT* pre-mRNA substrates additionally included α[^32^P]-ATP for internal radiolabeling. Transcribed spliceozyme and substrate *CAT* pre-mRNA were purified by 7 M urea 5% polyacrylamide gel electrophoresis (PAGE), eluted in 0.01% (w/v) SDS and 300 mM NaCl, ethanol precipitated, and redissolved in water. RNA concentrations were measured by their absorption at 260 nm.

### In vitro reactions

In vitro reactions were done essentially as described [22]. The final spliceozyme concentration was 1 µM or 100 nM, as noted, and *CAT* pre-mRNA substrate concentrations were 100 nM. Reactions were incubated in 5 mM MgCl_2_, 135 mM KCl, 50 mM MOPS/NaOH pH 7.0, 20 µM GTP, and 2 mM spermidine, at 37°C, in reaction volumes of 20 µL. Spliceozyme and substrate were pre-incubated separately in the reaction buffer for 10 minutes at 37°C before combining the solutions to start the reaction. Samples were taken in 2 µL aliquots after reaction times of 0, 1, 2, 5, 10, 20, 30, and 60 minutes. In vitro reaction products were separated on 7 M urea 6% PAGE and visualized by phosphoimaging. Bands were quantified on a phosphorimager (PMI, Bio-Rad) using the software Quantity One. The background was substracted using the rolling disk method, with two different methods to minimize artifacts. To quantify substrate and products, disk sizes were set such that the background-signal flanking each peak was contacted by the background subtraction line. To quantify the signal of the 5′-exon side product, the disk size was chosen larger such that the peaks of substrate and product were not separated. The signal strengths were then normalized for two factors: to take into account that each internally labeled fragment contained a different number of A's, the signal strengths of each fragment were divided by their number of A's. To account for gel loading errors, signals were also normalized for the total radioactivity per lane. This resulted in the molar fraction of substrate converted to product, which was reported in the figures. The time courses of the signal intensities were fitted for least-squares differences in Microsoft Excel using the Solver tool. All signals of products were fit well by single-exponential functions; all signals of substrates required double-exponential functions.

### Activity measurements in *E.coli*

Fresh overnight cultures of *E. coli* cells in LB medium containing 100 µg/mL ampicillin were diluted with LB medium to an A_600_ of 0.025, induced with IPTG at a final concentration of 1 mM, and shaken at 37°C for 1 hour. Growth was then measured on LB-agar plates instead of LB liquid culture because plates prevented single false-positive clones from overtaking the population [21]. Hundred µL of each IPTG-induced liquid culture were plated on one LB agar plate containing 100 µg/mL ampicillin. The same volume from the same cell suspension was plated on one LB agar plate containing the noted concentration of chloramphenicol and 1 mM IPTG. Plates were incubated at 37°C for 16 hours. Each plate was washed with 1.6 mL 1xPBS, and the A600 of each cell suspension was measured. The A_600_ obtained for LB-ampicillin plates was divided by the A_600_ from the corresponding LB-chloramphenicol plates to normalize the growth in the presence of chloramphenicol for the number of viable bacteria. The two introns used for the first activity measurements in *E. coli* cells (Figure 4B) were two clones resulting from the selection with 28 random nucleotides in the internal sequence of the intron. These two N_28_ sequences were 5′-GGCCACAGGCCCCGCGTCGCGGTGGGGC-3′ (clone 64-SiC2) and 5′-ATTCTTGATACTTTATTATTCAATTGTT-3′ (clone 64-SiC3).

### RT-PCR, sequence analysis, RT-qPCR, and plasmid yield

For the sequence analysis of in vitro splicing reactions, RNA was obtained by ethanol preparation after 60 minutes of incubation. For the analysis of in vivo splicing reactions, total RNA was isolated from logarithmically growing *E. coli* cultures using the Nucleospin RNA II kit (Machery-Nagel). In reaction volumes of 20 µL, RNA from the in vitro reaction containing 2 pmol *CAT* pre-mRNA and 20 pmol spliceozyme, or 3.2 µg of total RNA were incubated with 200 units of Superscript III reverse transcriptase (Invitrogen), for 60 minutes at 55°C, using the primer 5′-ccgtaacacgccacatc-3′ complementary to the *CAT* 3′ exon. DNA sequences were amplified by PCR with primers 5′-cggcctttattcacattct-3′ and 5′-gtgtagaaactgccggaa-3′. A nested, second PCR introduced restriction sites EcoRI and BamHI using primers 5′-gcatgaattccgcctgatgaatgctcat-3′ and 5′-gcatggatccgtattcactccagagcgat-3′ (restriction sites underlined). The products were cloned into plasmid pUC19b [21] and 10 clones were sequenced for each reaction.

To quantify the amount of *CAT* RNA 3′-exons in total RNA, reverse transcription products (see above) corresponding to 20 pg of total RNA were amplified in a volume of 10 µL using the two primers 5′-CCGTAACACGCCACATC-3′ and 5′-TGTTACACCGTTTTCCATGAG-3′, both of which anneal in the 3′-exon of the used *CAT* pre-mRNA sequence. The qPCR reactions were incubated with the Applied Biosystems qPCR master mix on the Fast 7500 RT-qPCR machine (Applied Biosystems), as described previously [21]. Only a single PCR product was observed in melting profiles.

Plasmid yields were determined by isolating plasmids from 2 mL of logarithmically growing *E. coli* cultures with an OD_600_ of 0.5, using the Nucleospin plasmid kit (Macherey Nagel). This was in the linear range of the assay, in which the plasmid yield from 2 mL bacterial culture correlated linearly with OD_600_ values in the range from 0.0 to 1.0 (data not shown).

### CAT activity assays

The CAT activity in *E. coli* cell lysate was determined essentially as described [35] but used a 4-fold higher number of cells per assay to obtain a strong signal. Cells from fresh overnight cultures were induced for one hour with 1 mM IPTG, diluted to an A_600_ of 0.02, and grown to an A_600_ of 0.20. Note that the promoters for *CAT* pre-mRNA and *CAT* mRNA were the same. Cells from two mL of culture were concentrated to 200 µL and frozen. After thawing, the suspensions were mixed with 200 µL of 200 mM Tris/HCl pH 7.8, 10 mM Na_2_EDTA, and 4 µL of toluene. Fifteen µL of this solution were diluted 10-fold with a buffer to obtain final concentrations of 1 mM DTNB, 0.2 mM Acetyl-CoA, and 0.2 mM chloramphenicol. The absorption at 412 nm was recorded in 15-second intervals. The slope of increase in the absorption at 412 nm was determined by linear least squares fitting to the readout between 6 minutes and 15 minutes. The lysate of cells expressing *CAT* mRNA without intron was diluted 10-fold to stay within the linear range of the assay. Units of CAT activity were determined using the extinction coefficient of 13,600 M^−1^ cm^−1^ for the reaction product, and the definition of one CAT unit, which corresponds to the acetylation of 1 µmol chloramphenicol per minute [35].

## Supporting Information

Figure S1 **Fraction of 5′-exon side products during the in vitro reaction of the spliceozyme.** The fraction is shown as the molar fraction of the *CAT* pre-mRNA at the start of the reaction (see Figure 2). (**A**) Influence of spliceozyme concentration and design at the ribozyme 5′-terminus. At the concentration of 1 mM (filled symbols) the 5′-exon was released 4-fold faster than at 100 nM (empty symbols). When the spliceozyme 5′-terminus ended in the P1 helix (squares) the release of the 5′-exon was 2-fold faster than when the spliceozyme 5′-terminus ended in the P1 extension (circles) or the 5′-duplex (triangles). (**B**) Influence of the length of the P9.2 duplex at the ribozyme 3′-terminus, with a 5′-terminal P1 duplex. The lengths of the P9.2 helix had no or a minor influence on the release of the 5′-exon, as judged by the comparison between P9.2 helices with a length of 9 base pairs (empty squares), 8 base pairs (empty diamonds), 7 base pairs (empty circles), 6 base pairs (empty triangles), and 5 base pairs (filled squares). Error bars show standard deviations from the average of triplicate experiments.
(PDF)
