# Supplementary material for: Spliceozymes: Ribozymes that Remove Introns from Pre-mRNAs in Trans
Source: PLoS One. 2014 Jul 11;9(7):e101932. doi: 10.1371/journal.pone.0101932 (PMC4094466; doi:10.1371/journal.pone.0101932)
Supplement: Figure S1 — Fraction of 5′-exon side products during the in vitro reaction of the spliceozyme. The fraction is shown as the molar fraction of the CAT pre-mRNA at the start of the reaction (see Figure 2). (A) Influence of spliceozyme concentration and design at the ribozyme 5′-terminus. At the concentration of 1 mM (filled symbols) the 5′-exon was released 4-fold faster than at 100 nM (empty symbols). When the spliceozyme 5′-terminus ended in the P1 helix (squares) the release of the 5′-exon was 2-fold faster than when the spliceozyme 5′-terminus ended in the P1 extension (circles) or the 5′-duplex (triangles). (B) Influence of the length of the P9.2 duplex at the ribozyme 3′-terminus, with a 5′-terminal P1 duplex. The lengths of the P9.2 helix had no or a minor influence on the release of the 5′-exon, as judged by the comparison between P9.2 helices with a length of 9 base pairs (empty squares), 8 base pairs (empty diamonds), 7 base pairs (empty circles), 6 base pairs (empty triangles), and 5 base pairs (filled squares). Error bars show standard deviations from the average of triplicate experiments. (PDF) [file pone.0101932.s001.pdf]

Figure S1

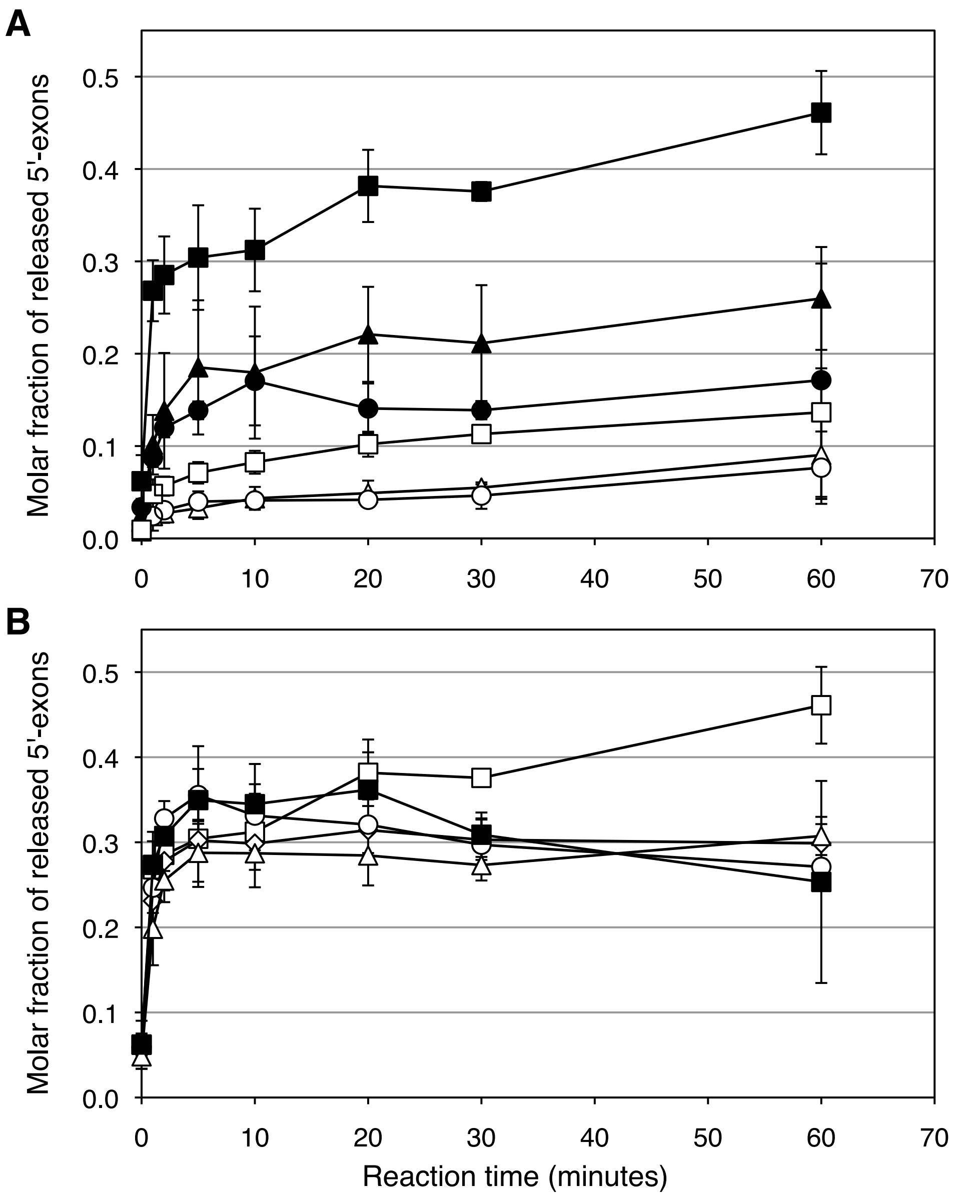

Figure S1. Fraction of 5'-exon side products during the in vitro reaction of the spliceozyme. The fraction is shown as the molar fraction of the *CAT* pre-mRNA at the start of the reaction (see figure 2). **(A)** Influence of spliceozyme concentration and design at the ribozyme 5'-terminus. At the concentration of 1 μM (filled symbols) the 5'-exon was released 4-fold faster than at 100 nM (empty symbols). When the spliceozyme 5'-terminus ended in the P1 helix (squares) the release of the 5'-exon was 2-fold faster than when the spliceozyme 5'-terminus ended in the P1 extension (circles) or the 5'-duplex (triangles). **(B)** Influence of the length of the P9.2 duplex at the ribozyme 3'-terminus, with a 5'-terminal P1 duplex. The lengths of the P9.2 helix had no or a minor influence on the release of the 5'-exon, as judged by the comparison between P9.2 helices with a length of 9 base pairs (empty squares), 8 base pairs (empty diamonds), 7 base pairs (empty circles), 6 base pairs (empty triangles), and 5 base pairs (filled squares). Error bars show standard deviations from the average of triplicate experiments.
